# Science and Math Interest and Gender Stereotypes: The Role of Educator Gender in Informal Science Learning Sites

**DOI:** 10.3389/fpsyg.2021.503237

**Published:** 2021-03-26

**Authors:** Luke McGuire, Tina Monzavi, Adam J. Hoffman, Fidelia Law, Matthew J. Irvin, Mark Winterbottom, Adam Hartstone-Rose, Adam Rutland, Karen P. Burns, Laurence Butler, Marc Drews, Grace E. Fields, Kelly Lynn Mulvey

**Affiliations:** ^1^Department of Psychology, University of Exeter, Exeter, United Kingdom; ^2^Department of Psychology, New York University, New York, NY, United States; ^3^Department of Psychology, North Carolina State University, Raleigh, NC, United States; ^4^Department of Educational Studies, South Carolina State University, Columbia, SC, United States; ^5^Faculty of Education, Cambridge University, Cambridge, United Kingdom; ^6^Department of Biological Sciences, North Carolina State University, Raleigh, NC, United States; ^7^Virginia Aquarium & Marine Science Center, Virginia Beach, VA, United States; ^8^Thinktank Science Museum, Birmingham, United Kingdom; ^9^EdVenture Children’s Museum, Columbia, SC, United States; ^10^Riverbanks Zoo & Garden, Columbia, SC, United States

**Keywords:** STEM interest, gender equity, gender stereotypes, informal science learning, science interest, math interest

## Abstract

Interest in science and math plays an important role in encouraging STEM motivation and career aspirations. This interest decreases for girls between late childhood and adolescence. Relatedly, positive mentoring experiences with female teachers can protect girls against losing interest. The present study examines whether visitors to informal science learning sites (ISLS; science centers, zoos, and aquariums) differ in their expressed science and math interest, as well as their science and math stereotypes following an interaction with either a male or female educator. Participants (*n* = 364; early childhood, *n* = 151, *M*^*age*^ = 6.73; late childhood, *n* = 136, *M*^*age*^ = 10.01; adolescence, *n* = 59, *M*^*age*^ = 13.92) were visitors to one of four ISLS in the United States and United Kingdom. Following an interaction with a male or female educator, they reported their math and science interest and responded to math and science gender stereotype measures. Female participants reported greater interest in math following an interaction with a female educator, compared to when they interacted with a male educator. In turn, female participants who interacted with a female educator were less likely to report male-biased math gender stereotypes. Self-reported science interest did not differ as a function of educator gender. Together these findings suggest that, when aiming to encourage STEM interest and challenge gender stereotypes in informal settings, we must consider the importance of the gender of educators and learners.

## Introduction

Girls and women often receive the message that the fields of science, technology, engineering and mathematics (STEM) are not “for them” (Furnham et al., 2002; Steele et al., 2002; Murphy et al., 2007). Gender-matched role models can challenge these inequitable ideas about STEM belonging (Marx and Roman, 2002; Stout et al., 2011; Master et al., 2014). Although teachers may act as role models (Master et al., 2014), youth also encounter STEM role models outside the classroom in informal science learning sites (ISLS; e.g., science centers, zoos, and aquaria). While interactions in ISLS are less likely to directly impact course enrolment or career trajectories, they may offer an important boost to STEM interest and challenge STEM gender stereotypes. The present study examines whether gender-matched educators are related to ISLS visitors’ interest and stereotypes in the domains of math and science.

### Promoting Interest

Promoting interest is important in fostering STEM engagement, motivation and future career aspiration (Kang et al., 2019). By 11–12-years-old, girls report lower STEM interest (Riegle-Crumb et al., 2011), especially in male-dominated subjects (Ceci and Williams, 2010; Diekman et al., 2010). At this age, children’s beliefs about their own ability are related to how they think about future careers (Bandura et al., 2001). Aspiring to a STEM career early on may be important, as students who have these career goals are more motivated to learn (Simons et al., 2004). Therefore, promoting interest should be a primary area of attention, as this may impact future career choice and current learning motivation.

Decreasing math interest amongst girls into adolescence has been well-documented (Frenzel et al., 2010) and is reflected in enrolment in mathematics courses (WISE, 2019). An understanding of math is central to advances in engineering, computing and the sciences (Wu, 2019) and reduced math interest is likely to impact engagement with these domains. Informal learning sites can bridge the gap between abstract mathematical concepts taught in school and their real-world applications (for example, through exhibitions on engineering). While science interest can decrease for girls between late childhood and adolescence (Vedder-Weiss and Fortus, 2010), these trends are more complex. For example, in the United Kingdom, while girls represent only 23% of students in advanced physics courses (WISE, 2019), there is little gender difference in chemistry and biology course participation (Cassidy et al., 2018). Given these different domain-specific trajectories, a focus on science and math interest offers insights into the differential effects of gender-matched mentors within ISLS.

### Identity-Based Motivation in STEM

Disparities in gender representation within STEM are consistent with the cues that men naturally “belong” in these fields. Identity-based motivation (IBM; Oyserman and Destin, 2010) theory argues that identities can act as motivation, with individuals being more likely to engage in behaviors that feel congruent with their identities. Crucially, IBM theory suggests that identities and behaviors are contextually and dynamically constructed. For example, in the context of ISLS, if a girl witnesses an example of STEM expertise demonstrated by a female educator (for example, interacting with a female educator guiding an activity focused on biology in the zoo context), she may then come to believe that success in STEM can be a component of her female identity.

If contextual cues can lead to the dynamic construction of identity, then it is likely that the presence of educators also contributes to this identity construction process. Researchers have examined whether interactions with formal educators who challenge STEM gender stereotypes limit the negative consequences of gender stereotypes for STEM engagement among females. This research has most often been conducted in formal educational contexts (Dee, 2006). For example, female adolescents are less concerned about being negatively stereotyped when their teacher is female than when their teacher is male (Master et al., 2014). Further, when asked to describe a role model who inspired them to pursue a particular career or educational path (including, but not limited to, STEM careers), female participants more frequently described a female role model (Lockwood, 2006). So far, we know less about the benefits of children’s interactions with counter-stereotypical STEM educators outside of formal contexts, especially within informal science education settings (ISLS).

These ISLS act as important environments for learning (Andre et al., 2017) where knowledgeable educators can scaffold children’s learning (Bamberger and Tal, 2007). However, less is known about how educator identities interact with children’s own identities to influence their interest in STEM in ISLS. One study demonstrated that adolescents who participated in a weekend science program at a science center referenced increased science interest due to interactions they had with educators (Price et al., 2019). An important next step is to understand whether shared gender with the ISLS educator is related to visitors’ interest and their stereotypes about who usually succeeds in STEM.

Given the importance of female role models for female students in formal education settings, we tested the hypothesis that math and science interest would differ based on the gender of the educator and the gender of the participant. Specifically, we expected that girls would report higher math and science interest following an interaction with a female educator, compared to an interaction with a male educator (Hypothesis 1). This was expected due to the dynamic construction of identity as outlined in IBM theory. We anticipated that seeing a female educator demonstrate their STEM expertise would allow girls to see STEM expertise as part of the broader female identity and in turn lead to a boost in their interest. Evidence suggests that the greatest drop in interest occurs between late childhood and adolescence (Frenzel et al., 2010). Therefore, we anticipated that the “protective” effect of a female educator would be most pronounced prior to this age range (i.e., late childhood) (Hypothesis 2). In contrast, we did not expect differences in math and science interest as a function of educator gender for boys, given their membership a group whose identity is already aligned with STEM gender stereotypes.

Second, we expected that responses to math and science gender stereotype measures would differ depending on the gender of the educator with whom participants interacted, and the gender of the participant. Specifically, we expected that girls would be less likely to report damaging male-biased STEM stereotype responses (i.e., perception that boys rather than girls or both girls and boys are usually good at math/science) when they interacted with a female educator, compared to when they interacted with a male educator (Hypothesis 3). As per Hypothesis 1, we based this hypothesis on IBM theory and the idea that encounters with female STEM expertise would allow female visitors to bring the idea of STEM expertise within their conception of female identity and in turn to challenge male-biased stereotypes. Again, we did not expect differences in reporting of male-biased stereotypes based on educator gender for boys, given the congruence of their identity with STEM gender stereotypes.

## Methods

### Participants

Three-hundred forty-six participants (female *n* = 219) were recruited from four ISLS in the United Kingdom and the United States. In the United Kingdom, participants visited a science museum in the Midlands (*n* = 84). In the United States, participants were visitors to an aquarium (*n* = 123), a zoo (*n* = 82), and a children’s science museum (*n* = 57), all based in the Southeast. Participants were divided into three age groups: early childhood (*n* = 151, *M*^*age*^ = 6.73, *SD* = 1.12), late childhood (*n* = 136, *M*^*age*^ = 10.01, *SD* = 0.80), and adolescence (*n* = 59, *M*^*age*^ = 13.92, *SD* = 2.16). Sixty-four percent of participants identified as members of the ethnic majority group of the country (United Kingdom: White British United Kingdom; United States: White European American). See Supplementary Material for full sample ethnicity demographics.

### Procedure

In the United States, all measures were approved by the University of South Carolina Department of Educational Studies IRB as part of the “STEM Teens” project. In the United Kingdom, all measures were approved by the Ethics Committee of the Goldsmiths, University of London Psychology Department as part of the same project. The protocol was completed using either online survey software (Qualtrics, Provo, UT, United States) on a tablet computer, or in hard copy using the same measures. Parental consent and child assent were obtained for all participants.

The measures described below were part of a questionnaire examining youth STEM engagement. The survey included measures of gender and ethnic stereotyping, STEM engagement and interest, content questions and self-reported learning. These measures were counterbalanced and the order in which participants completed them was randomized. An experimenter explained the procedure to families. In order to ensure understanding, experimenters read the survey with younger children who were unfamiliar with using a tablet or less confident in reading ability. Older children capable of using a tablet and confident in reading ability completed the survey alone, with a researcher present to offer clarifications.

Participants were recruited as part of family groups (at least one adult and one child) and offered either an electronic gift card, gift shop voucher or gift bag (worth $/£5) for participation. Participants were approached following an interaction with an educator around static media or cart activities. These interactions most often involved educators guiding children’s learning through the use of learning aids (e.g., showing children a gorilla skull at a zoo exhibit about Gorillas), assisting with exhibition activities (e.g., explaining how a circuit board works in an exhibition about a car), or answering questions about the exhibit topic not covered by static media. Eighty-two percent of participants reported their educator interaction lasted 5 min or less. Visitors interacted with either male (*n* = 151) or female (*n* = 195) educators based upon ISLS’ own scheduling of educators, rather than at random.

## Measures

### Math and Science Interest

Participants were asked “How interested are you in math?” and “How interested are you in science?” (1 = *not at all interested*, 5 = *really interested*).

### Math and Science Stereotype Awareness

Using two questions adapted from Liben and Bigler (2002), participants were asked “Who is usually good at math?” and “Who is usually good at science?” and asked to select “boys” (male-biased response), “girls” (female-biased response), or “both boys and girls” (equitable response).

### Data Analytic Plan

Although we did not expect differences across data collection locations, we calculated intra-class correlation coefficients (ICC) across sites, and across exhibits within sites. For science interest (site ICC = 0.02; exhibit within site ICC = 0.03), math interest (site ICC = 0.04; exhibit within site ICC = 0.04), and stereotype response (site ICC = 0.003, exhibit within site ICC = 0.004) low coefficients suggested multi-level modeling was not required.

Participants’ math and science interest responses were subjected to 3 (participant age; early childhood, late childhood, and adolescence) × 2 (participant gender; female, male) × 2 (educator gender; female, male) ANOVAs. Pairwise comparisons were carried out with Bonferroni corrections for multiple comparisons.

Because our hypotheses focused on gender stereotypes and identity congruence, we examined whether participant age, gender and educator gender predict whether participants showed more or less male-biased stereotype responses. In order to do so, a dummy variable for Male Stereotype Bias was created (1 = participant responded “boys” usually good at math/science, 0 = participant responded “girls” or “both boys and girls” usually good at math/science). Binary logistic regression models assessed whether age, participant gender, or educator gender were related to participants’ male-biased stereotype responses. Age, participant gender, and educator gender were entered in step one. At step two, two interaction terms were added: first between participant gender and educator gender, and second between participant age and participant gender.

## Results

### Math Interest

Analyses revealed a main effect of participant gender, *F*(1, 317) = 5.71, *p* = 0.02, η*^2^* = 0.02. Boys (*M* = 3.94, *SD* = 1.39) reported greater math interest than girls (*M* = 3.62, *SD* = 1.44). There was also a main effect of participant age group, *F* (2, 317) = 8.92, *p* < 0.001, η*^2^* = 0.05. Participants in early childhood (*M* = 4.01, *SD* = 1.37) reported greater math interest than participants in late childhood (*M* = 3.67, *SD* = 1.42, *p* = 0.04) and adolescence (*M* = 3.22, *SD* = 1.46, *p* = 0.006). There was no difference between math interest in late childhood and adolescence. There was no main effect of educator gender on math interest (*p* = 0.30).

**FIGURE 1:**
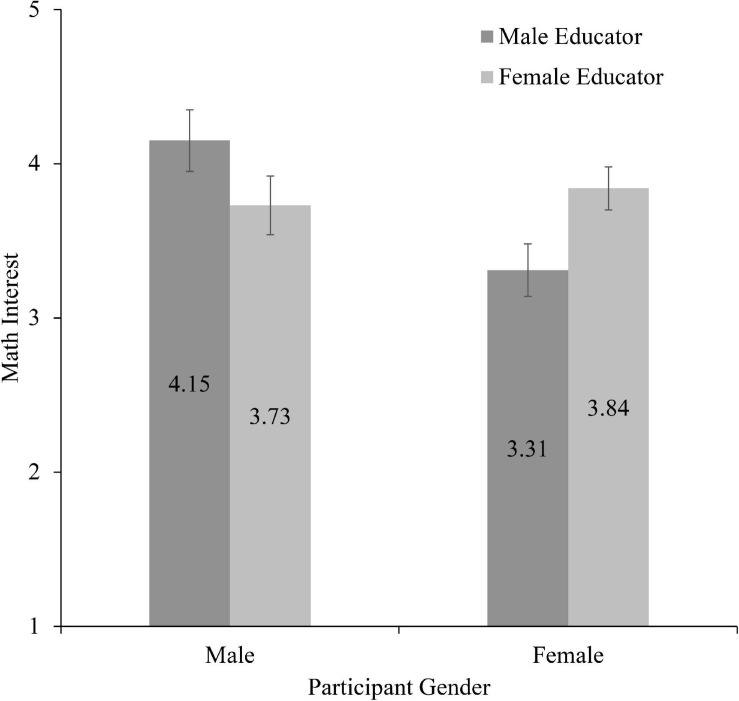
Math interest as a function of participant gender and educator gender (w. standard error bars).

In support of Hypothesis 1, analyses revealed a significant interaction between participant gender and educator gender, *F*(1, 317) = 10.48, *p* = 0.001, η*^2^* = 0.03 (see Figure 1). Pairwise comparisons suggested that when girls interacted with a female educator (*M* = 3.84, *SD* = 1.38) they reported greater math interest than when they interacted with a male educator (*M* = 3.31, *SD* = 1.48, *p* < 0.001). For boys there was no difference in math interest based on whether they interacted with a female educator (*M* = 3.73, *SD* = 1.48) or a male educator (*M* = 4.15, *SD* = 1.26, *p* = 0.30). Further, when comparing between participant gender groups, girls reported lower math interest following an interaction with a male educator compared to boys who interacted with a male educator (*p* < 0.001). In contrast, there was no difference in math interest between boys and girls who interacted with a female educator (*p* = 0.40).

Counter to Hypothesis 2, participant age group did not interact with either participant gender (*p* = 0.80) or educator gender (*p* = 0.06), nor was there a three-way interaction between these variables (*p* = 0.40). For girls, interacting with a female educator is associated with greater self-reported math interest than a similar interaction with a male educator.

### Math Gender Stereotype Awareness

**FIGURE 2:**
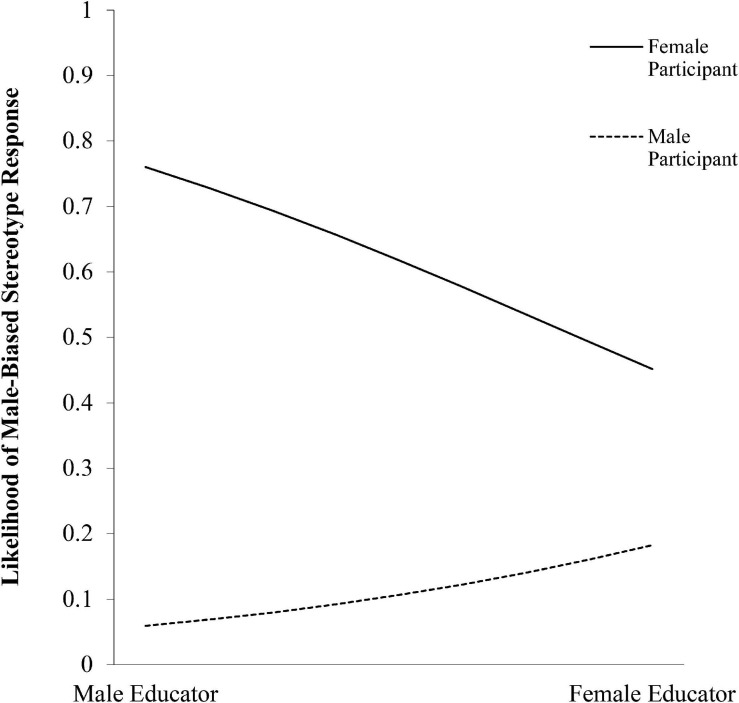
Likelihood of male-biased stereotype response as a function of participant gender and educator gender.

Overall, 58% of participants responded that “both boys and girls” were usually good at math. At step one, the model was significant, *X*^2^(3) = 13.40, *p* = 0.004, Nagelkerke *R*^2^ = 0.06. Girls were more likely than boys to respond that boys were usually good at math, β = 0.91, Wald (1) = 11.13, *p* = 0.001. Neither participant age nor educator gender were related to participants’ male-biased math gender stereotype responses. At step two, the model was significantly improved by adding the interaction term between participant gender and educator gender, *X*^2^(5) = 20.30, *p* = 0.001, Nagelkerke *R*^2^ = 0.09. Girls who interacted with a female educator were less likely to respond that boys were usually good at math, compared to when they interacted with a male educator, β = −1.31, Wald (1) = 5.66, *p* = 0.02 (Figure 2). The interaction between participant age and participant gender was not significant, β = 0.12, Wald (1) = 1.38, *p* = 0.24.

In support of Hypothesis 3, girls reported greater interest in math and were less likely to give male-biased responses to a math stereotype awareness measure following an interaction with a female educator (compared to a male educator).

### Science Interest

**FIGURE 3:**
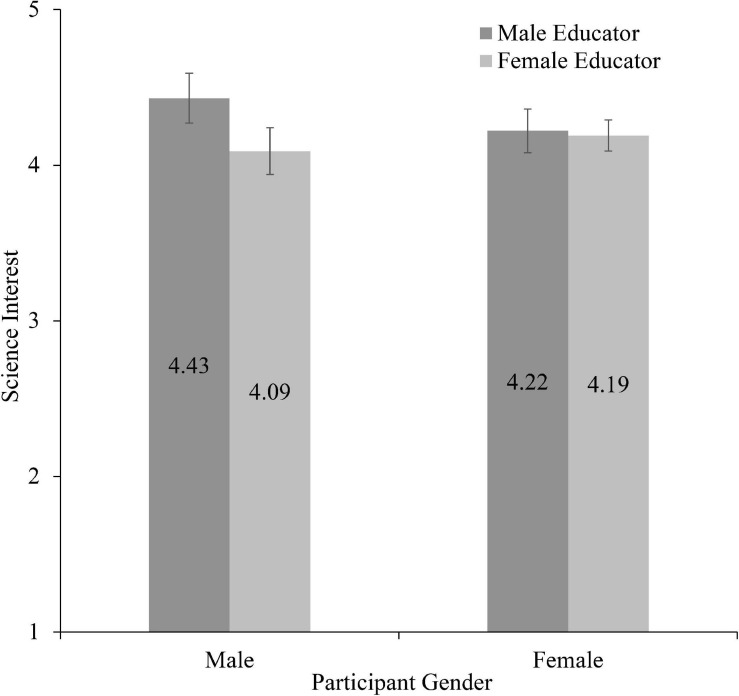
Science interest as a function of participant gender and educator gender (w. standard error bars).

We did not observe main effects of participant age (*p* = 44), participant gender (*p* = 0.56), or educator gender (*p* = 0.23) on participants’ self-reported science interest (see Figure 3). Further, we did not observe significant two-way interactions nor a three-way interaction between these variables.

### Science Gender Stereotype Awareness

Overall, 72% of participants responded that “both boys and girls” were usually good at science. At step one, the model was significant, *X*^2^(3) = 23.72, *p* < 0.001, Nagelkerke *R*^2^ = 0.11. Girls were more likely than boys to respond that boys were usually good at science, β = 1.11, Wald (1) = 12.34, *p* < 0.001. Age was negatively related to male-biased stereotype responses, β = −0.18, Wald (1) = 7.49, *p* = 0.006. Educator gender was not related to participants’ gender stereotype responses. At step two, while the model was significant *X*^2^(4) = 24.05, *p* < 0.001, Nagelkerke *R*^2^ = 0.12, neither of the interaction terms significantly predicted whether participants gave more male-biased stereotype awareness responses (see Table 1).

**TABLE 1:** Logistic regression analysis of children’s male-biased math stereotype awareness responses.

**Predictor**	**β**	**SE β**	**Wald’s χ^2^**	***df***	***p***	**eβ (odds ratio)**
Age	–0.06	0.05	1.30	1	0.25	0.94
Gender (1 = female, 0 = male)	0.91	0.27	11.13	1	0.001	0.40
Educator Gender (1 = female, 0 = male)	–0.04	0.27	0.02	1	0.89	0.97
Gender (1) by Educator Gender (1)	–1.31	0.55	5.66	1	0.02	3.59
Gender (1) by Age	0.12	0.10	1.38	1	0.24	1.62

Educator gender was not related to visitors’ self-reported interest in science, nor did it impact their male-biased science stereotype responses.

## Discussion

The findings demonstrate a role for educator gender in relation to math interest and stereotypes as measured in ISLS: while most participants reported egalitarian responses to our stereotype measures, girls who interacted with a female educator (compared to a male educator) reported greater math interest and were less likely to report that only boys were usually good at math. In contrast, self-reported science interest did not vary as a function of either participant gender, age group or educator gender. These findings have implications for matching visitor and educator gender in ISLS.

In line with previous research, girls reported lower interest in math than boys (Blickenstaff, 2005; Riegle-Crumb et al., 2011). However, girls reported higher math interest when they interacted with a female educator compared to girls who interacted with a male educator, and as compared to male participants who interacted with a male educator. One possible explanation for this effect is that female STEM educators are counter-stereotypical and thus interacting with and sharing their expertise in ISLS challenges the belief that STEM subjects are only for males. In line with IBM theory (Oyserman and Destin, 2010) female educators can offer contextual examples of STEM expertise, allowing female visitors to dynamically construct their math identity, and perhaps see math as more closely aligned with their own identity. In contrast, interacting with a male educator may reaffirm the belief that math expertise is not a core part of the female identity, therefore reducing self-reported interest in this area.

In line with recent research (McGuire et al., 2020) math gender stereotype responses were predominantly equitable (i.e., 58% reported that “both boys and girls” are usually good at math). However, when participants did not choose this option, we observed differences depending on educator gender. Specifically, girls who interacted with a female educator were less likely to report that only boys were usually good at math. This suggests that a match between visitor and educator gender is important for increasing interest in math, but also as a buffer against male-biased gender stereotype responses. As IBM may suggest, as girls interact with different educators throughout their visit, their ideas of whether STEM is for “someone like me” are likely to change depending on whether they see their own identity reflected in the educational staff they meet.

Longitudinal research is required to determine the longevity of any positive buffering effect following an interaction with a female educator. One possibility is that this effect is similar to that of the stereotype-lift or stereotype boost effect (Walton and Cohen, 2003) and may not extend beyond the scope of the particular study context. Research has documented that implicit gender stereotypes are related to the stereotype lift effect (Franceschini et al., 2014). One possibility is that girls in the present study who have weaker implicit gender stereotypes are more likely to benefit from interactions with female educators. Future research ought to examine stereotypes before and after interactions with educators in order to more accurately assess the impact of such interactions.

Counter to our predictions, we did not observe differences in self-reported science interest or gender stereotypes based on educator gender, participant gender, or age group. Science interest was high across all our sample whereas in contrast math interest was lower on average. Therefore, one possibility is that there was simply more room to build upon baseline math interest compared to high science interest. This would make sense given that our participants were visitors to science focused ISLS. Similarly, 72% participants reported that “both boys and girls” were usually good at science. Again, this is perhaps not surprising since our visitors to ISLS were accompanied by parents or guardians who actively choose to visit these science-oriented sites. While participants did not differ in terms of self-reported science interest, female participants *were* more likely to report that boys are “usually” good at science. Contrary to the domain of math, interactions with a female educator did not offer a buffer against this male-biased stereotype response.

Although we know that long-term interactions in formal settings can protect against the effects of STEM stereotype threat (Marx and Roman, 2002; Master et al., 2014), it appears that the strength of male-biased stereotypes about science are less likely to be challenged by short-form interactions in ISLS. Prior studies using short-form interactions have shown no effect of gender matching role models on enhancing science motivation in adolescence (Hoffman and Kurtz-Costes, 2019). Further, the sites in which we tested were diverse (e.g., science museum as compared to aquarium). Despite small ICCs suggesting multi-level modeling was not necessary, it is possible that this diversity coupled with asking about “science” broadly (rather than domain-specific questions about physics compared to biology, for example) may contribute to the egalitarian stereotype responses lack of educator effects. Further research is essential to understand how conditions of informal interactions (e.g., intersection with other identities, length of interaction, forging of personal connection) may offer examples of identity-congruent expertise that *can* challenge stereotypes.

The effects of interacting with a female educator held for girls across late childhood and adolescence. We anticipated that the buffering effects of a female educator would be most beneficial for an age-range where a drop in interest has been previously documented (i.e., in late childhood; Ceci and Williams, 2010; Riegle-Crumb et al., 2011). However, it appears that the stereotype disconfirming effects of an educator apply across this developmental span and even earlier in childhood. This is further evidence that informal STEM experiences are invaluable not just for learning, but also for challenging ideas about who can engage with STEM.

### Limitations and Future Directions

Here, single item measures were used for timeliness of data collection. Future work should use scale measures of math and science interest. For example, validated instruments of science interest (Lamb et al., 2012), or the Children’s Math Interest Self Report measure (Fisher et al., 2012) could provide further evidence of the relation between educator gender and interest. Further complementing self-report measures by probing whether interest effects translate into behavioral differences is crucial; for example, do participants who report greater interest also persist for longer on relevant interactive tasks? While coherence between youth and educator gender is related to interest, we do not yet know the mechanism that underlies this effect. Future research could measure whether interacting with an educator from the participants’ own gender is related to an increase in identification with science or math, which in turn may be related to increased interest and engagement.

The ISLS in which we collected data were predominantly focused on science, and yet our results demonstrated differences in math interest. One possibility is that visitors generalize from observing expertise in science into the related domain of math. Future work could be conducted to test this possibility by comparing against a non-STEM subject (e.g., language, arts). We would expect to see differences in math interest when girls interact with a female educator in a science context, but not differences in non-STEM interest. This would provide evidence that observing expertise in one STEM area can transfer to interest in other related domains, rather than just a general boost in interest in all subjects. Further, a more granular approach to examination of the science domain is essential in order to understand whether demonstrations of expertise in our sites (for example, physical science expertise in the science museum) translates in to challenges to gender stereotypes about science areas where gender inequality is greater and gender stereotypes more strongly founded.

Finally, an important possibility for future research is to examine the effects of interacting with an ethnic identity-congruent expert. Research has documented that visitors from ethnic-minority groups feel that ISLS are not “for me” (Dawson, 2014). The presence of expert educators who share ethnic identities with visitors to these sites may offer one important route to challenging stereotypes and broadening conceptions of who belongs in these sites.

## Conclusion

This study demonstrates that matched interactions based on gender are important in informal learning sites. For girls, ISLS represent an opportunity to interact with female STEM experts who not only provide a buffer against male-biased stereotypes but may also promote STEM interest. STEM experiences outside of the classroom are not only relevant for learning, but also for challenging male-biased ideas about ability and enhancing interest and motivation.

## Data Availability Statement

The datasets generated for this study are available on request to the corresponding author.

## Ethics Statement

The studies involving human participants were reviewed and approved by Goldsmiths, University of London Psychology Department, and the Department of Educational Studies at the University of South Carolina. Written informed consent to participate in this study was provided by the participants’ legal guardian/next of kin.

## Author Contributions

LM: acquisition, analysis, interpretation of data, drafting first full version of manuscript, and revised drafts. KLM, MI, MW, AR, and AH-R: conception and design of work, interpretation of data, and critical evaluation of draft manuscript. TM: analysis of data and critical evaluation of draft manuscript. FL and AH: critical evaluation of draft manuscript. KB: conception of work, acquisition of data, final approval of manuscript. LB, GF, and MD: acquisition of data, final approval of manuscript. All authors contributed to the article and approved the submitted version.

## Conflict of Interest

The authors declare that the research was conducted in the absence of any commercial or financial relationships that could be construed as a potential conflict of interest.
