# Supplementary material for: Science and Math Interest and Gender Stereotypes: The Role of Educator Gender in Informal Science Learning Sites
Source: Front Psychol. 2021 Mar 26;12:503237. doi: 10.3389/fpsyg.2021.503237 (PMC8033013; doi:10.3389/fpsyg.2021.503237)
Supplement: Supplementary file 1 [file Table_1.DOCX]

Supplemental Table 1: Sample Ethnicity

| Ethnicity | Frequency | Percentage |
| --- | --- | --- |
| African-American | 36 | 10.1 |
| American Indian/Native American | 5 | 1.4 |
| Asian/Asian-American | 15 | 4.2 |
| Bi-Racial/Multi-Racial (US) | 16 | 4.5 |
| Black British | 2 | 0.6 |
| Hispanic/Latinx | 9 | 2.5 |
| Mixed Race/Dual-Heritage (UK) | 5 | 1.4 |
| Native Hawaiian/Pacific Islander | 5 | 1.4 |
| Other (UK) | 4 | 1.1 |
| Other (US) | 16 | 4.5 |
| Pakistani British | 5 | 1.4 |
| White British | 63 | 17.6 |
| White/European American | 165 | 46.2 |
